# Synthesis-Dependent Magnetic Modifications in Starch-Coated CoFe_2_O_4_ Monodomain Nanoparticles: Structural, Magnetic and Spectroscopic Study

**DOI:** 10.3390/nano15191504

**Published:** 2025-10-01

**Authors:** Zorica Ž. Lazarević, Valentin N. Ivanovski, Aleksandra Milutinović, Marija Šuljagić, Ana Umićević, Jelena Belošević-Čavor, Ljubica Andjelković

**Affiliations:** 1 Institute of Physics—National Institute of the Republic of Serbia, University of Belgrade, P.O. Box 68, Pregrevica 118, Zemun, 11080 Belgrade, Serbia; miluta@ipb.ac.rs; 2 Department of Nuclear and Plasma Physics, Vinča Institute of Nuclear Sciences—National Institute of the Republic of Serbia, University of Belgrade, P.O. Box 522, 11001 Belgrade, Serbia; valiva@vin.bg.ac.rs (V.N.I.); umicev@vin.bg.ac.rs (A.U.); cjeca@vin.bg.ac.rs (J.B.-Č.); 3 Institute of Chemistry, Technology and Metallurgy, Department of Chemistry, University of Belgrade, 11000 Belgrade, Serbia; marija.suljagic@ihtm.bg.ac.rs

**Keywords:** CoFe_2_O_4_, starch functionalization, Raman spectroscopy, Mössbauer spectroscopy

## Abstract

This study investigates the structural and magnetic properties of CoFe_2_O_4_ nanoparticles prepared by five different synthesis methods: coprecipitation, ultrasound-assisted coprecipitation, coprecipitation coupled with mechanochemical treatment, microemulsion and microwave-assisted hydrothermal synthesis. The produced powders were additionally functionalized with starch to improve biocompatibility and colloidal stability. The starch-coating procedure itself by sonication in starch solution, as well as its result, affects the structural and magnetic properties of functionalized nanoparticles. The resulting changes of properties in the process of ligand addition depend significantly on the starting nanoparticles, or rather, on the method of their synthesis. The structural, magnetic and spectroscopic properties of the resulting materials were systematically investigated using X-ray diffraction (XRD), Raman spectroscopy, Mössbauer spectroscopy and magnetic measurements. Taken together, XRD, Raman and Mössbauer spectroscopy show that starch deposition reduces structural disorder and internal stress, resulting in nanoparticles with a more uniform size distribution. These changes, in turn, affect all magnetic properties—magnetization, coercivity and magnetic anisotropy. Magnetic responses are preserved what is desirable for future biomedical applications. This work emphasizes the importance of surface modification for tailoring the properties of magnetic nanoparticles while maintaining their desired functionality.

## 1. Introduction

Ferrites, particularly cobalt ferrite nanoparticles (CoFe_2_O_4_), have proven to be transformative materials in the biomedical field due to their unique magnetic properties and biocompatibility when properly functionalized. Cobalt ferrite (CoFe_2_O_4_) belongs to the spinel ferrite family with the general formula AB_2_O_4_, in which divalent (A^2+^) and trivalent (B^3+^) cations occupy tetrahedral (A) and octahedral (B) sites in a cubic close- packed oxygen framework. The degree of cation distribution between these sites strongly influences the structural, magnetic and electronic properties of spinels [1,2,3]. In particular, CoFe_2_O_4_ exhibits high magneto crystalline anisotropy and moderate saturation magnetization, chemical stability and mechanical hardness, making it a model system for fundamental studies and for applications in data storage [4,5], catalysis [6,7] and spintronics [8,9]. Cobalt ferrite magnetic nanoparticles demonstrate considerable versatility in medical imaging applications by serving as contrast agents for magnetic resonance imaging and enabling better visualization in computed tomography scans [10,11,12,13]. Beyond diagnostic applications, ferrite nanoparticles offer promising therapeutic opportunities through targeted drug delivery systems, where their magnetic properties allow precise navigation to specific tissues under external magnetic field guidance [13,14,15,16,17]. The hyperthermia treatment capabilities of ferrites represent another promising approach. They enable the local heating of tumor tissue through the application of alternating magnetic fields, providing a minimally invasive cancer treatment option [18].

Despite their potential, ferrite nanoparticles face inherent problems related to their high surface-to-volume ratio, which promotes agglomeration tendencies. Such magnetic nanoparticles, characterized by hydrophobic surfaces and dipolar interactions, exhibit strong interparticle attraction leading to cluster formation and subsequent particle enlargement.

The inherent reactivity and potential toxicity of pure ferrite nanoparticles requires careful surface functionalization to ensure biocompatibility and prevent agglomeration in biological environments. Surface functionalization strategies include two primary approaches: ligand exchange and ligand addition [19,20]. In ligand exchange, the original surface is replaced by specific functional groups such as thiol, carboxylic acid, amine or polydopamine moieties, thereby improving the surface properties. Conversely, ligand addition is based on the adsorption of polymers on the surfaces of nanoparticles through hydrophobic interactions, electrostatic forces and hydrogen bonds. The polymers used can be synthetic materials such as polyethylene glycol (PEG), polyacrylic acid (PAA), polyvinylpyrrolidone, polyvinyl alcohol and polymethacrylic acid or natural polymers such as chitosan, starch, cellulose, agarose and dextran. The integration of natural polymers, especially polysaccharides such as starch, through ligand addition mechanisms solves these challenges by providing biocompatible surface coatings that retain magnetic functionality while reducing toxicity and improving colloidal stability [19,20]. This combination of magnetic sensitivity, therapeutic potential and biocompatible surface modification positions ferrite nanoparticles as essential components in next-generation biomedical applications, from advanced diagnostic imaging to targeted therapeutic interventions.

**Figure 1:**
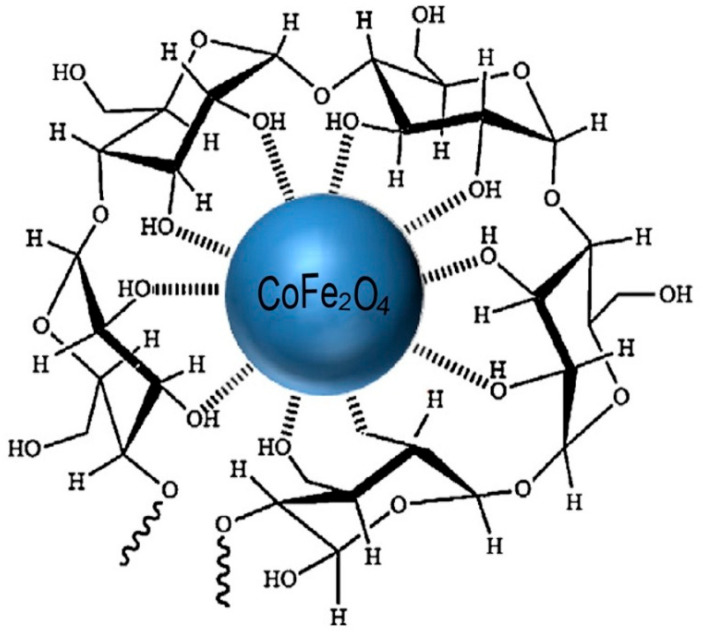
Schematic illustration of chemical structure of starch-coated CoFe_2_O_4_ nanoparticle.

The structural analysis, magnetic anisotropy evaluation, nanoparticle size distribution assessment and blocking temperature determination of pure cobalt ferrites have been studied in detail [21,22]. This work focuses on the comprehensive structural analysis of starch-coated monodomain nanoparticles of partially inverse cobalt ferrite, (Co_1-x_Fe_x_)^A^[Fe_2-x_Co_x_]^B^O_4_, prepared by five different synthesis methods: co-precipitation (CO), ultrasound-assisted co-precipitation (US-CO), co-precipitation followed by mechanochemical treatment (MC-CO), microwave hydrothermal synthesis (MW-HT) and microemulsion (ME). In the surface functionalization of CoFe_2_O_4_, the method of ligand addition was used, in which starch molecules attach to the surfaces of the nanoparticles by hydrogen bonding with the hydroxyl groups on the surface of the oxide materials (Figure 1) [23]. The study is complemented by Raman and Mössbauer spectroscopic investigations of both the bare and coated nanomaterials. The aim of these in-depth investigations is to develop optimal synthesis protocols to produce starch-coated CoFe_2_O_4_ nanoparticles with the desired properties.

## 2. Materials and Methods

The following reagents were purchased from Sigma-Aldrich (St. Louis, MO, USA) (analytical grade purity) and used as received: Iron (III) chloride hexahydrate (FeCl_3_·6H_2_O, 98%), cobalt (II) chloride hexahydrate (CoCl_2_·6H_2_O, 98%), sodium hydroxide (NaOH, >97%), cetyltrimethylammonium bromide (CTAB, >98%), n-butanol (99.8%), n-hexanol (>99%), iron(III) nitrate nonahydrate (Fe(NO_3_)_3_·9H_2_O, >99.95%), cobalt(II) nitrate hexahydrate (Co(NO_3_)_2_·6H_2_O, >99.99%), ammonium hydroxide solution (28% NH_3_ in water), absolute ethanol, and soluble starch. Distilled water was used throughout experimental procedures [21].

Several synthesis strategies were utilized to prepare the CoFe_2_O_4_ nanoparticles, including coprecipitation (conventional, ultrasonically-assisted and mechanochemically-treated variants), microwave hydrothermal processing and microemulsion-based preparation [21].

### 2.1. Coprecipitation Synthesis

The material was prepared by dissolving 0.02 mol Fe^3+^ and 0.01 mol Co^2+^ chloride salts in 50 mL deionized water, then heating to the boiling point. Complete precipitation of the metal cations was achieved by rapid addition of excess 1 M NaOH solution with constant stirring, resulting in the formation of a black precipitate (pH of solution was 11). The reaction mixture was refluxed for 1 h, then cooled to room temperature and filtered. The precipitate was washed with distilled water until the hydroxide was completely removed (neutral pH), then separated by vacuum filtration and dried at 100 °C for 2 h. The resulting powder was divided equally into two portions: One fraction was ground with an agate mortar and calcined in an electric furnace (SNOL 3/1100 LHM01, Utena, Lithuania) (heating rate: 10 °C min^−1^) at 450 °C for 1 h, while the second portion was subjected to a planetary grinding process.

### 2.2. Mechanochemical Synthesis

The treatment by ball milling was carried out using a planetary mill (PM100CM, Retsch GmbH, Düsseldorf, Germany) equipped with a hardened-steel vessel (500 cm^3^ capacity) containing ten hardened-steel balls (8 mm diameter). The powder obtained by coprecipitation was ground for 10 h at 500 rpm in sealed hardened steel containers with a mass ratio of 20:1 between ball and sample. The support disc and vessel operated at angular velocities of 32.2 and 40.3 rad s^-1^, respectively, producing a grinding intensity equivalent to approximately 10-fold gravitational acceleration.

### 2.3. Ultrasonically-Assisted Coprecipitation Synthesis

CoCl_2_·6H_2_O and FeCl_3_·6H_2_O solutions were combined in stoichiometric ratios, with Fe^3+^ and Co^2+^ concentrations of 0.4 M and 0.2 M, respectively, in a final volume of 50 mL. An excess of 1 M NaOH was added rapidly with constant stirring, maintaining the pH of the solution at 11. The mixture was heated to 80 °C and sonicated approximately 1 h. The precipitated particles were washed extensively with deionized water to remove hydroxide residues and impurities and then filtered. The precipitate was dried at 80 °C, ground in an agate mortar, and annealed in an electric furnace following the same protocol as the coprecipitation sample.

### 2.4. Microemulsion Synthesis

A microemulsion system was prepared from CTAB (surfactant), n-butanol (co-surfactant), n-hexanol (oil phase), and an aqueous solution (aqueous phase). The composition of microemulsion comprised 15 wt% hexanol, 45 wt% aqueous solution, and 60/40 surfactant-to-cosurfactant ratio. Two identical microemulsions were prepared with different aqueous phases: one with stoichiometric Fe^3+^ and Co^2+^ nitrate solutions, and another containing ammonium hydroxide as precipitant. These microemulsions were combined under constant stirring and heated at 90 °C for 1 h, reaching a pH value of about 11. The product-containing microemulsion was treated with a water/ethanol mixture and separated by centrifugation. The precipitate was washed with absolute ethanol to remove residual oil and surfactant, isolated by vacuum filtration, and dried at 70 °C. After grinding in an agate mortar, the material was annealed in an electric furnace under the same conditions as previously described powders.

### 2.5. Microwave-Assisted Hydrothermal Synthesis

The stoichiometric amounts of Fe^3+^ and Co^2+^ chloride salts required for the preparation of 1.5 g CoFe_2_O_4_ were dissolved in deionized water, then a slight excess of ammonium hydroxide was added to achieve pH value of about 10. The total volume of the mixture was divided into seven 100 mL vessels, each containing 50 mL of the solution. The vessels were positioned in a segmented high-pressure rotor HPR-1000/10S (MILESTONE, Milan, Italy) and processed in a microwave stove (ETHOS 1, Advanced Microwave Digestion System, MILESTONE, Milan, Italy). Microwave power ranged from 0–1000 W with linear heating at 20 °C min^−1^, followed by 200 °C treatment for 20 min under maximum 100 bar pressure. After cooling in air, the particles were separated by vacuum filtration and repeatedly washed with deionized water to remove the excess of chlorides. The synthesized nanoparticles were dried at 70 °C for 24 h, then ground in an agate mortar.

### 2.6. Functionalization with Starch

Each CoFe_2_O_4_ powder (0.5 g) was ultrasonically dispersed in 20 mL deionized water. Separately, 2.5 g of starch was dissolved in boiling water, and 200 mL of a 2 M NaOH solution was prepared. The starch solution and the ferrite dispersion were combined and added dropwise to the hydroxide solution. The resulting mixtures were sonicated at 80 °C for 1 h and then cooled to room temperature. The samples were washed several times with small amounts of deionized water until the neutral pH of the filtrate solution was reached in order to remove the excess of starch and hydroxide. Then, samples were separated by vacuum filtration, and dried at ambient temperature for 48 h.

### 2.7. Characterization Techniques and Magnetic Measuerements

The X-ray diffractograms were recorded using an automated Rigaku SmartLab diffractometer (Rigaku Corporation, Tokyo, Japan) with Cu Kα source. The diffraction angle range was 15–90° 2θ with a step of 0.01° and a scanning speed of 2° min^−1^.

The micro-Raman spectra were collected by a triple Jobin Yvon T64000 spectrometer (Horiba Ltd., Kyoto, Japan) configured for backscattering geometry. This system is equipped with a nitrogen-cooled CCD detector (Horiba Ltd., Kyoto, Japan). Coherent Verdi G optically pumped semiconductor laser perating at 532 nm output laser power of about 20 mW was used as an excitation source for recording Raman spectra in the range from 100 cm^−1^ to 1000 cm^−1^. The Raman scattering measurements on nanopowders were performed at room temperature using an objective with 50× magnification and a numerical aperture of 0.75 to focus the laser on a spot with a diameter of about 1 mm. To avoid localized heating of the sample surface by the laser irradiation, neutral density filters were used to transmit 10 or 1% of the incident light to further reduce the laser power to less than 1 mW at the input of the spectrometer’s optical system. Each exposure lasted about 2 min.

The ^57^Fe-Mössbauer spectra of the starch-coated CoFe_2_O_4_ samples were obtained at ambient temperature in the velocity range of ~±12 mm/s. The spectra were recorded in standard transmission geometry in constant acceleration mode with a ^57^Co(Rh) source. The Mössbauer spectra were calibrated using natural iron spectrum. The Recoil program [24] was used to fit the measured spectra. The values of center shift (*CS*) are quoted relative to the natural iron (*CS* = 0).

The magnetic measurements were performed with a commercially available Quantum Design Physical Property Measurement System (PPMS) equipped with a 9 T superconducting magnet and a vibrating sample magnetometer (VSM) option (Quantum Design, San Diego, CA, USA)). The temperature dependence of the magnetization, M (T), was measured from 5 to 300 K when heated in the field-free (ZFC) and field-cooled (FC) regimes at 100 Oe. The hysteresis loops, *M* (H), were measured at 300 K in the field range ±9 T.

## 3. Results and Discussion

### 3.1. XRD

**Figure 2:**
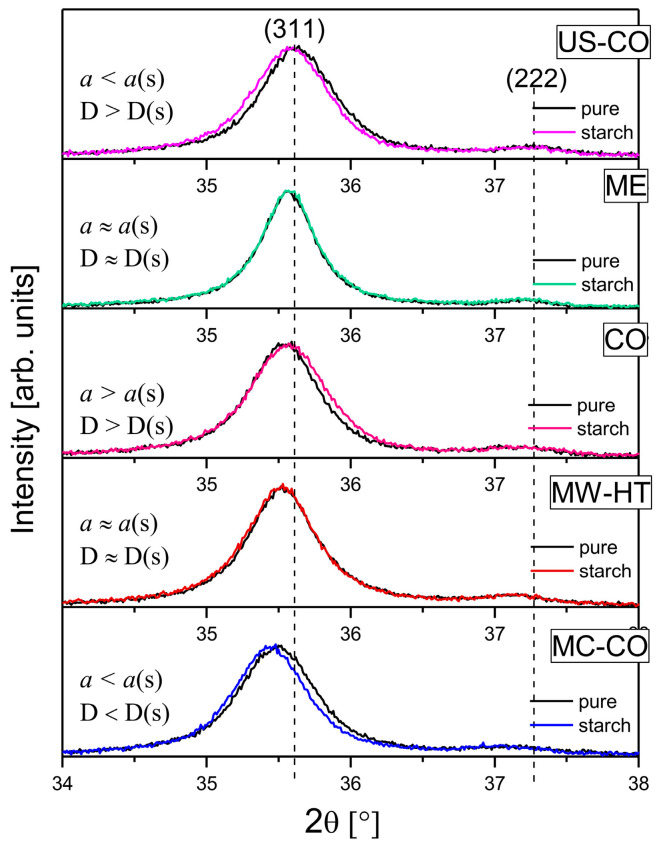
A detail of normalized diffractograms of bare and starch-coated CoFe_2_O_4_ nanoparticles with the strongest Bragg reflection (311): it is evident that the smaller nanoparticles US-CO, CO and MC-CO (with broader (311) reflections) underwent greater changes during the starch coating process. Auxiliary dashed line coincides with the center of the (311) reflection of the US-CO sample with the smallest lattice constant. Diffractograms are in order of ascending lattice constant.

The XRD analysis was conducted to find out how the coating process changes the structural properties of the synthesized cobalt ferrites. To compare bare nanomaterials and their coated counterparts, the results of XRD analysis from previous work is presented [22]. Figure 2 shows 2 θ region from 34° to 38° of the normalized X diffractograms of the uncoated (bare) and starch-coated nanomaterials with (311) Bragg reflection. It can be clearly seen that the smaller nanoparticles US-CO, CO and MC-CO, with broader (311) reflections, underwent major changes during the starch coating process. Careful analysis of the diffractograms of coated nanoparticles provided quantitative data on the effect of the coating process itself on the starting nanomaterials.

**Figure 3:**
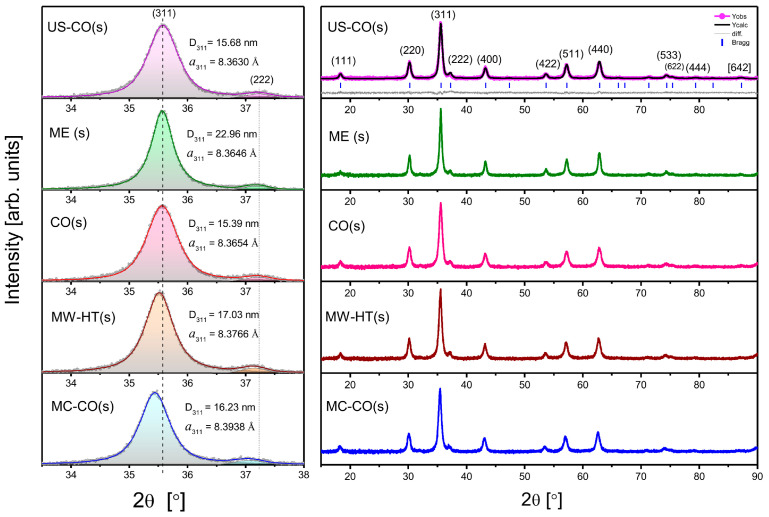
X-diffractograms of starch-coated CoFe_2_O_4_ samples: Left is part of spectra with the strongest Bragg reflection (311). Auxiliary dashed line coincides with the center of the (311) reflection of the US-CO(s) sample with the smallest lattice constant. Diffractograms are in order of ascending lattice constant.

The X-ray diffractograms of the starch-coated CoFe_2_O_4_ nanopowders are shown in Figure 3, in the 2θ range from 15° to 90°. Starch-coated CoFe_2_O_4_ samples are labeled with an abbreviation indicating the synthesis method used (as for the bare samples), with the letter “s” for starch added in parentheses. All diffractograms were analyzed by Rietveld profile refinement using *FullProf Suite,* Version: September 2020 OriginPro 2016.. The diffraction peaks were simulated with the *Pseudo-Voigt* function. The same function is used to fit the strongest (311) Bragg peak with the aid of *OriginPro 2016*, Figure 3, left. The lattice constant *a*_311_ is estimated using the well-known Laue–Bragg’s relation for cubic crystals:

*a*_hkl_ = λ·(h^2^ + k^2^ + l^2^)^1/2^/[2·sinθ_hkl_] (1)

The average nanoparticle size, *D*_311_, is calculated by Debye–Scherrer relation:

*D*_hkl_ = 0.94·λ/[(w−w_i_)_hkl_·cosθ_hkl_] (2)

where the instrumental FWHM (Si standard sample), w_i_, for about 35.5°, is 0.07°.

With a higher starch content, a broad hump would be visible in the 10°–25° regions of the diffractograms [25]. Figure 3 shows only well-crystallized CoFe_2_O_4_ with the space group *Fd*$\bar{3}$*m* (No. 227).

Table 1 lists the structural parameters of the CoFe_2_O_4_ samples calculated from the diffractograms by Rietveld profile refinement and from the strongest peak (311). In the first row for each nanomaterial are values determined for bare CoFe_2_O_4_ samples [22]. In the second row are the values for the starch-coated samples. The corresponding differences between coated and uncoated samples are given in brackets.

It is known that the refinement of the diffractogram according to Rietveld is not sufficiently sensitive for the inversion of cations whose atomic factors are of similar size. Therefore, XRD structure analysis was used to estimate more reliable values for cation inversion.

**Figure 4:**
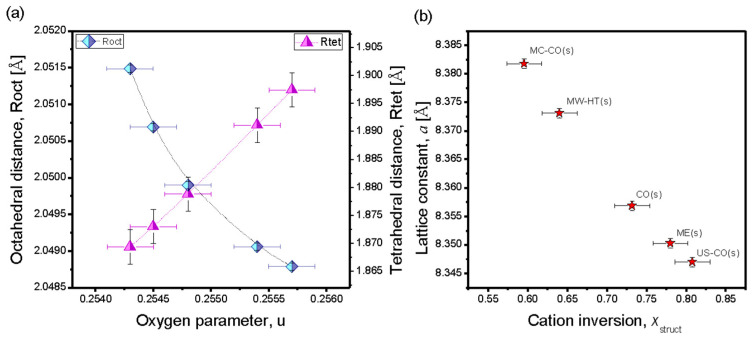
XRD structural analysis of starch-coated NP-samples. (**a**) *R*_oct_ and *R*_tet_ are presented as functions of u; (**b**) lattice constant as function of *x*_struct_. Absolute error obtained from XRD structure analysis for calculated cation inversion is ±0.02.

Figure 4 shows the results of the XRD structural analysis of the NP samples. The octahedral (*R*_oct_) and tetrahedral (*R*_tet_) distances, as well as the cation inversion (*x*_struct_) are estimated via Equations (3)–(6), which relate the lattice parameters that were accurately ordered by the Rietveld profile analysis: Lattice constant (*a*_exp_) and oxygen parameter (u) with known Shannon ionic radii [26]. The effective radius of the Co^2+^ cation with a high spin (*HS*) electron arrangement in tetrahedral position is *r*(Co^2+^)^A^ = 0.58 Å, and in octahedral position the radius is *r*(Co^2+^)^B^ = 0.745 Å. For Fe^3+^ in tetrahedral position the effective radius is *r*(Fe^3+^)^A^ = 0.485 Å (HS) and in octahedral position the radius is *r*(Fe^3+^)^B^ = 0.645 Å. The oxygen radius is *r*_O_ = 1.38 Å.

*R*_tet_ = <*r*_A_> + *r*_O_ = (1 − *x*)·*r*(Co)^A^ + *x·r*(Fe)^A^ + *r*_O_ (3)

*R*_oct_ = <*r*_B_> + *r*_O_ = 1/2·[(2 − *x*)·*r*(Fe)^B^ + *x·r*(Co)^B^] + *r*_O_ (4)

When the unit cell origin is B—vacancy (like in *FPSuite* programme), ideal $u^{\bar{3}m}$ = ¼ and the cation to anion distances are given by:

(5) $$R_{\mathrm{tet}}\;=\;a_{\exp}\;\sqrt{3}(u-1/8)$$

(6) $$R_{\mathrm{oct}}=a_{\exp}\;\sqrt{\left(3u^{2}-2u+3/8\right)}$$

In Figure 4a *R*_oct_ and *R*_tet_ are presented as functions of *u*, and in Figure 4b the lattice constant is shown as a function of *x*_struct_. The migration of larger Co^2+^ cations from B sites to A sites, i.e., the decrease in inversion, leads to an increase in the lattice constants (and all hopping distances, which depend only on *a_exp_*). The obtained values of the cationic inversion, *x_struct_*, are listed in Table 1.

The analysis of the XRD spectra, Table 1, shows that the starch deposition process led to a decrease in the size of the magnetic CoFe_2_O_4_ nanoparticles of the US-CO(s) sample, a decrease in the cation inversion coefficient and an increase in the lattice constant. The reduction in the internal stress confirms the presence of a starch coating, which could lead to a reduction in the internal stress at least in the magnetic shell of the nanoparticles (and thus also to a reduction in the average stress).

US-CO(s) is the only sample that showed a significant decrease (Δ*x*_struct_ < 0) in the inversion coefficient, so a noticeable increase in magnetization can be expected.

The lattice constant of the coated nanomaterial ME(s) remains the same as that of the pristine material. The cation inversion coefficient also remains unchanged (Δ*x*_struct_ ≈ 0), but the size of the nanoparticles increases slightly due to the coating process. The increase in nanoparticles and even a minimal island-like coating lead to a decrease in the internal stress.

In the case of the starch-coated CO(s), the average size of the nanoparticles is significantly smaller and consequently the internal stress is higher. As a result of the increase in internal stress, a contraction of the crystal lattice (Δ*a* < 0) occurred.

At first glance, it appears that the MW-HT(s) sample was not affected by the starch coating process. But a slight decrease in particle size and a slight decrease in internal stress confirm that starch coating has taken place.

MC-CO(s) has an increase in lattice constant, Δ*x*_struct_ ≈ 0, a small increase in particle size, but a significant decrease in internal stress, indicating a very good starch coating.

### 3.2. Magnetic Properties

**Figure 5:**
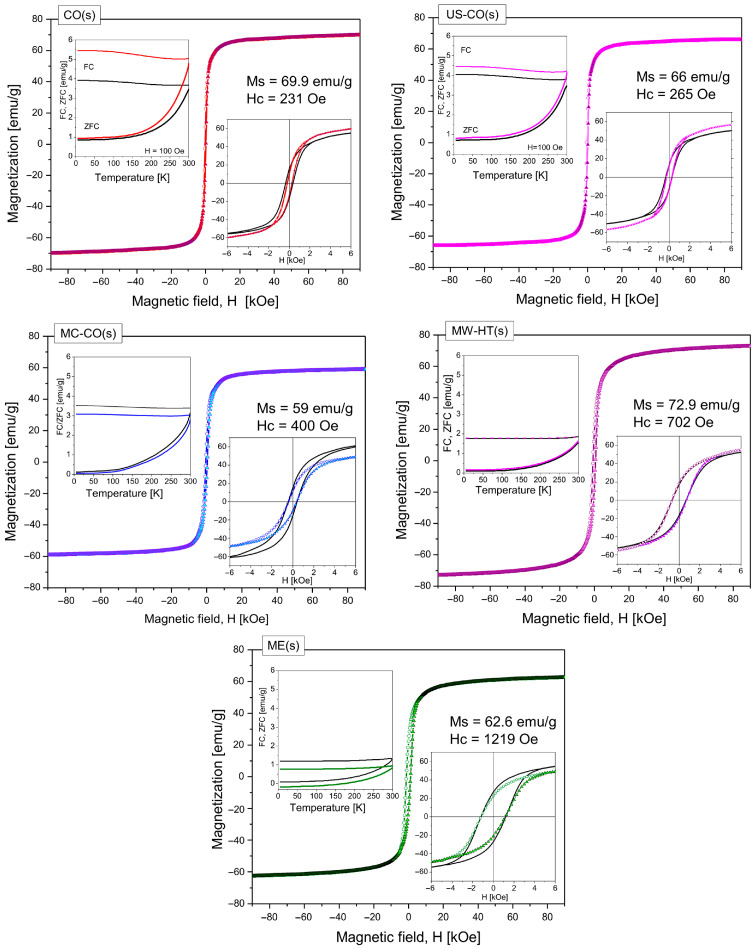
Magnetic measurements of cobalt ferrite nanomaterials obtained by various synthesis methods and coated by starch. In the central part of each graphic is magnetization as the function of the magnetic field in the range ±90 kOe. In the right insert are hysteresis loops of pure nanomaterial (black line) and the same nanomaterial after starch coating (line + symbol in color); in the left insert there is the ZFC/FC measurement (under warming) in the range of 5–300 K. Black lines indicate pure nanomaterial, color lines indicate starch-coated. Above hysteresis loops for all samples are given saturation magnetization *M*_s_ and coercivity field *H*_c_ for starch-coated nanoparticles. Samples are in order of ascending coercivity and descending values of FC (at 5 K) magnetization.

The results of magnetic measurements of starch-coated cobalt ferrite nanoparticles obtained by various synthesis methods are shown in Figure 5. To make it easier to see the changes that occurred during the starch coating process, along with the magnetic hysteresis of the starch-coated nanomaterials, the hysteresis loops of the pure counterparts are also given. The same was applied to the results of FC/ZFC magnetization measurements. The left insets of the graphs for certain nanomaterials show the magnetizations of both the pure and coated counterparts together. All data were recorded during heating from 0 to 300 K in a weak magnetic field, *H* = 100 Oe. Magnetization FC (*M*_FC_) was measured after cooling in the presence of the same field. Magnetization ZFC (*M*_ZFC_) was measured after previous cooling in the absence of a field. The separation magnetizations of FC and ZFC as well as their curvatures indicate that the blocking temperatures *T*_B_ are below 300 K, see ref. [22]. This means that in all ensembles of the investigated CoFe_2_O_4_ nanopowders a larger proportion of superparamagnetic particles is present at a measurement temperature of *M = f(H)*. Due to the presence of a large number of SP nanoparticles, at higher fields, beyond the hysteresis region, all samples have an asymptotic value of magnetization. For magnetic measurements at 300 K, CoFe_2_O_4_ nanoparticles smaller than 8 nm are in the superparamagnetic state in a time interval of about 100 s [27]. In Figure 5, the samples are arranged in order of increasing coercivity and decreasing FC magnetization values.

The measured (in *H* = ±90 kOe) and calculated magnetic characteristics of starch-coated monodomain CoFe_2_O_4_ nanopowders obtained by different synthesis methods are given in Table 2. The coercivity, *H*_c_, in the case of single-domain nanoparticles increases significantly with the size of the magnetic nanoparticles. It can be seen that as the coercivity increases, the “squareness” also increases, i.e., the magnetization curves become steeper. This is an expected trend and we have already seen it with these nanomaterials before their coating with starch [22].

What changed strikingly after starch coating was the increased values of the slope of the magnetization curves of the smallest CO(s) and US-CO(s) nanoparticles in the *H* = 0 region. (Corresponding slopes for bare nanoparticles were 13% lower for CO (46.7 emu g^−1^/kOe) and 18% lover for US-CO (43.7 emu g^−1^/kOe), as reported in Ref. [22]). The increase in the rate of tracking changes in the magnetic field indicates a decrease in the interaction between the particles. The reason for this could be a decrease in the average size of the nanoparticles (which was confirmed by XRD). This increases the proportion of the surface layer (shell), which has a lower magnetic order. With a decrease in the average size, the proportion of superparamagnetic particles in the ensemble may also increase. Another possible reason for the lower interaction between the particles is their coating with starch. Depending on the amount of adsorbed starch and the type of coating (complete or partial shielding of the magnetic core), the effects can be different. In the case of MC-CO(s) nanomaterials, the starch coating led to a reduction in the influence of the magnetic field on the magnetic core of the nanoparticles. The decrease of the magnetization slope in the region close to the zero-field shows that the starch content in the MC-CO(s) nanopowders is significant. In general, starch coating leads to an increase in structural order in the magnetically depleted shell under the starch.

In the high field range, *H*⟶90 kOe, the slopes of magnetization of US-CO(s) and MC-CO(s) have drastically decreased. For the bare US-CO nanoparticles the slope of magnetization was 0.037 (about 40% higher) and for MC-CO it was 0.035 emu g^−1^/kOe (24% higher) [22]. This clearly indicates a better structural order and a reduction in the number of canting spins due to the thickness coating. The magnetization slopes of larger nanoparticles did not show such correlated and significant changes.

**Figure 6:**
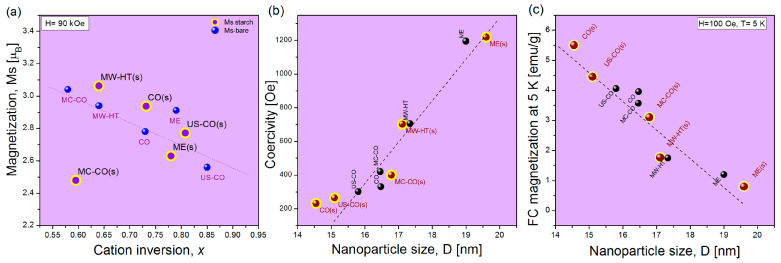
(**a**) Room temperature saturation magnetization at *H* = 90 kOe of bare (solid blue circles) and starch-coated (blue circles with yellow edge) nanoparticles as a function of cation inversion; (**b**) coercivity of bare (black circles) and coated NPs (brown circles with yellow edge) as a function of size; (**c**) magnetization *M*_FC_ at *T* = 5 K and *H* = 100 Oe of bare and starch-coated NP-s as a function of size.

A brief discussion of the results of magnetic measurements of pure cobalt ferrite nano materials [22] and their starch-coated counterparts, in light of the outcomes of the XRD analysis, is presented in Table 3 and Figure 6.

Since the coercivity (*H*_c_) of single-domain CoFe_2_O_4_ nanoparticles is approximately proportional to the size of the magnetic particles [22], it can be assumed that in cases where the particle size decreases during the starch deposition process (Δ*D* < 0), a decrease in coercivity (Δ*H*_c_ < 0) also occurs, and vice versa.

For well-coated NPs, a reduction in magnetization proportional to the amount of capping material is expected [25]. For nanoparticles where TGA has confirmed a significant amount of starch, such as US-CO (s) and MC-CO (s), a decrease in magnetization is expected. However, for the US-CO (s) sample with Δ*x*_struct_ < 0, the increase in magnetization was not reduced by the deposited starch. It is greater and corresponds to *x* = 0.81. This means that the starch is not uniformly deposited (it is probably island-like) and magnetic core of nanoparticles is not completely encapsulated.

The magnetization of the MC-CO(s) sample was significantly reduced, by up to 18%. This indicates a uniform deposition of starch in a fairly thick layer.

In the case of ME(s) with a much lower amount of starch, the reason for the reduction of *M_s_* could be the capping of the primary aggregates (not individual nanoparticles). In this case, a lower amount of starch would be sufficient. Aggregates of larger nanoparticles with stronger magnetic moments may be more resistant to disintegration by stirring in a starch solution. In general, the primary aggregates are disassembled (partially or completely) and different aggregates of nanoparticles are formed. The different agglomeration of the NPs could be the reason for higher values of magnetization *M*_s_ in CO(s) and MW-HT(s) [27].

The results of magnetic measurements of CoFe_2_O_4_ nanomaterials before and after functionalization with starch are illustrated in Figure 6 as a function of structural parameters determined by Rietveld and XRD structural analysis. The saturation magnetization, *M_s_*, is given as a function of the coefficient of cation inversion *x*_struct_ (obtained from XRD structural analysis), Figure 6a. The coercivity measured at 300 K and magnetization *M*_FC_ at 5 K (obtained from FC/ZFC measurements) are shown as a function of the size of the nanoparticles (estimated from the Williams–Hall plot) in Figure 6b,c, respectively.

In Figure 6a are shown saturation magnetizations of bare and starch-coated CoFe_2_O_4_ nanomaterials. *M*_s_ of bare nanoparticles regularly decreases with increasing of cation inversion and the dashed line corresponds to the linear fit of *M*_s_ = *f*(*x*) for bare nanomaterials (*μ*_Fe_ = 3.25 *μ*_B_ and *μ*_Co_ = 2.5 *μ*_B_) [22]. Magnetization of small NP-s dominantly depends on the cation inversion, but at the same time, *M*_s_ depends on the size of nanoparticles. As can be seen, larger ME nanoparticles with better structure, have higher *M*_s_ than is expected for their coefficient of the cation inversion.

The magnetization of the starch-coated samples shows the effect of starch deposition and the consequences of the mixing process in the starch solution. Changes in the magnetization that are not accompanied by corresponding changes in Δ*x* are the result of altered agglomeration of the magnetic nanoparticles. Therefore, only significantly reduced values of Δ*M* < 10% (which cannot be associated with an increase in *x*) undoubtedly confirm the success of the encapsulation process, i.e., that the nanoparticles or their agglomerates are completely coated with a layer of starch. A larger amount of starch results in a smaller contribution from the magnetic core and a lower total magnetization of the coated nanoparticles. Additionally, diamagnetic starch partially reduces the total magnetization of the coated nanoparticle because its own magnetization is oriented opposite to the external magnetic field.

The coercivity *H*_c_ = *f*(*D*) is proportional to the size of the nanoparticles, Figure 6b. For all coated samples, *H*_c_ is less than 2–43%, except for ME(s) where it is 1% higher. The decrease in *H*_c_ values for the starch-coated samples indicates less interaction between the particles (due to the presence of starch) and consequently easier realignment in an external magnetic field. In addition to the influence of starch, the *H_c_* value is also influenced by the size change that may have taken place during the starch deposition process. The increase in nanoparticle size due to the increased core/shell ratio leads to an improvement in the structure (on average), and thus to an increase in mutual interactions and a more difficult realignment of magnetic moments in an external magnetic field. The increase in ME(s) nanoparticles can be one reason for the increase in Hc. On the other hand, the CO(s) sample has the lowest *H*_c_ value (43% lower than *H*_c_ of pure CO). This decrease in *H*_c_ confirms that the average size of the NPs in the CO(s) sample is significantly reduced.

The starch coating process has probably led to the partial dissolution of small nanoparticles due to the strong mixing of the solution. Then, Ostwald ripening can lead to the growth of larger nanoparticles at the expense of the reduction of smaller nanoparticles.

The decrease in *H*_c_, (which is barely perceptible on the broad scale of *H*_c_ in Figure 6b, despite the increase in NP size for some nanomaterials (except CO(s)), shows that even incomplete capping has reduced the dipole magnetic interaction in the NPs.

The value of the magnetization in a weak magnetic field, *H* = 100 Oe, at 5 K (and the curvature of the magnetization *M*_FC_ = *f*(*T*)) depends on the magnetic anisotropy as *H*_c_. For pure samples, *M*_FC_ = Const. − *f*(*D*), (black dashed line) at *T* = 5 K, i.e., the decrease in *M*_FC_ is proportional to the increase in particle size *D*, Figure 6c. The *M*_FC_ values of the starch-coated samples show the same trend as for the pure nanomaterials. There are no comparable changes to the magnetization measured at 300 K, Figure 6a. A significantly larger value of the magnetic moments of the cations in the nanoparticles of CoFe_2_O_4_ at 5K (*μ*_Fe_ = 5 *μ*_B_ and *μ*_Co_ = 3.36 *μ*_B_) is observed compared to *μ*_Fe_ = 3.25 *μ*_B_ and *μ*_Co_ = 2.5 *μ*_B_ at 300 K [22], overcoming the influence of the covering starch layer at low temperatures. The graph in Figure 6c shows the changes in size of the magnetic CoFe_2_O_4_ nanoparticles before and after the starch coating process, but without any visible effect of the starch itself on the values of *M*_FC_.

To summarize, a relatively small amount of starch is bound to the NP-s, so the starch had no effect on reducing agglomeration. In TEM and SEM [21], no starch is visible. Perhaps new aggregates of nanoparticles were formed due to the incomplete coating of starch.

The starch coating should reduce the surface tension and thus the internal tension to a certain degree. This effect was observed for all coated nanoparticles, except for CO(s), where a significant reduction in NP size caused an increase in internal tension. If the starch had completely covered the surfaces of the NPs, the effect would have been more pronounced, so that an increase in the lattice constants could have occurred, but an increase in *a*_exp_ is only visible for US-CO(s) and MC-CO(s).

In the case of CO(s), *a*_exp_ decreased. Partial dissolution of a large proportion of the small particles in CO(s) occurred, so that the average size in the ensemble decreased, the internal stress increased and the crystal lattice contracted.

In MC-CO(s), a sufficiently large amount of starch is registered on the surface of the nanoparticles, which leads to the obscured of the magnetic core and a significant decrease in the saturation magnetization, *M*_s_.

The 10% decrease of *M*_s_ in ME(s) is difficult to explain due to the extremely low amount of adsorbed starch (if the coating of primary aggregates is excluded). At the same time, a slight increase in *H_c_* and *D* was registered.

### 3.3. Raman Spectroscopy

As we have already seen, all analyzed CoFe_2_O_4_ samples showed a typical XRD pattern of *Fd-3m* space group, i.e., macroscopically are cubic. The factor group analysis predicts 42 phonon modes for the normal cubic spinel structure: three acoustic of *T*_1u_ symmetry and 39 optic modes distributed among the following symmetries in the center of the Brillouin zone [28,29]:

*Γ* = *A*_1g_(R) + *E*_g_(R) + 3*T*_2g_(R) + 4*T*_1u_(IR) + *T*_1g_ + 2*A*_2u_ + 2*E*_u_ + 2*T*_2u_ (7)

Five of these phonon modes are Raman active, namely *A*_1g_, *E*_g_ and 3*T*_2g_; four are IR active, 4*F*_1u_, and remaining modes are silent.

**Figure 7:**
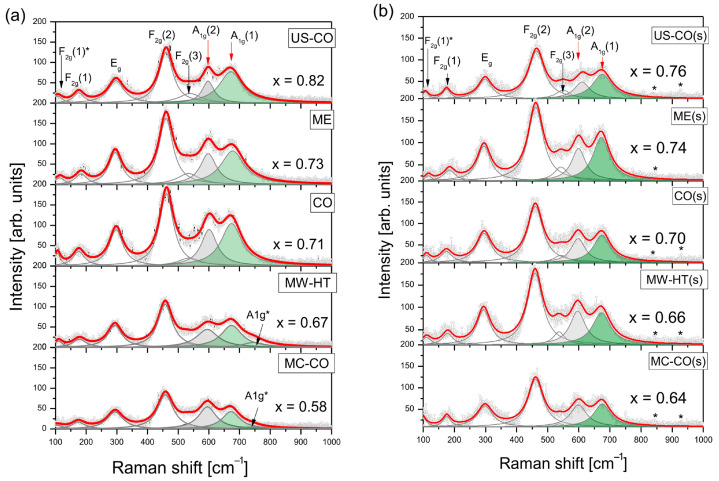
(**a**) Raman spectra of bare nanoparticles CoFe_2_O_4_ produced by various synthesis routes; (**b**) Raman spectra of starch-coated nanoparticles. *—possible modes of starch.

Raman spectra are more sensitive to local symmetry and exhibit asymmetric or dissociated peaks characteristic of inverse and partially inverse spinel structures. For simplicity (as is usual), the Raman modes are assigned as for normal cubic spinel. Figure 7 shows the Raman spectra of bare and starch-coated nanopowders with fully separated components of the *A*_1g_ mode typical for (Co_1-_*_x_*Fe*_x_*)[Fe_2-*x*_Co*_x_*]O_4_.

The spectra of the coated NPs do not clearly show the presence of starch, probably due to the low amount of starch and the poor quality of the spectra. The starch peaks around 480, 860 and 940 cm^−1^ are not visible, but small bumps at 840 cm^−1^ and 930 cm^−1^ in some spectra, marked with an asterisk in Figure 7b, could be a trace of starch [25].

Raman spectra are fitted with seven (or eight) Lorentzians. The values of the wavenumbers are generally consistent with the literature [30].

The *A*_1g_ mode is divided into an *A*_1g_ (1) component, which arises from the stretching vibrations of O^2-^ in tetrahedra with central cation Fe^3+^ (Fe^3+^-O4 bonds), and an *A*_1g_(2) component, which arises from the stretching vibrations of O^2-^ in tetrahedra with cation Co^2+^ (Co^2+^–O4 bonds). The intensities of these modes are proportional to the number of corresponding cations in the A-site of the cubic spinel, so that the value of the inversion coefficient *x*_R_ can be roughly estimated from the ratio of the intensities:

*x*_R_ = *I*_A1g(1)_/[(*I*_A1g(1)_+ *r·I*_A1g(2)_)] (8)

Here, *r*—stands for the relative oscillator strength of the Co^2+^-O_4_ bonds in relation to the Fe^3+^-O_4_ bonds and *I*_A1g(1,2)_ are the corresponding Lorentzian areas. The force constant *k*, which determines the mode frequency *ω*^2^ ∝ *k*/m, where m is the reduced mass of the ions, is directly proportional to the charges Z_A_ and Z_O_ the central cation or oxygen of the A site and inversely proportional to the cubic number of the associated bond length *r*_A-O_, as *k* ∝ Z_A_Z_O_/r_A-O_^3^ [31]. The ionic radii of the tetrahedral Co^2+^ and Fe^3+^ ions are 0.58 Å (high spin) and 0.49 Å (high spin), respectively, and the radius of the oxygen O^2-^ in the tetrahedra is 1.38 Å [26]. Therefore, the relative oscillator strength of the Co^2+^ bonds in relation to the Fe^3+^ bonds in tetrahedra is:

*r* = [Z_Co_/Z_Fe_]/[r_Fe-O_^3^/r_Co-O_^3^] = 2/3 (0.49 + 1.38)^3^/(0.58 + 1.38)^3^ = 0.579 (9)

The calculated Fe contents in the tetrahedral range, *x*_R_, for various cobalt ferrite samples are shown in Figure 7.

**Figure 8:**
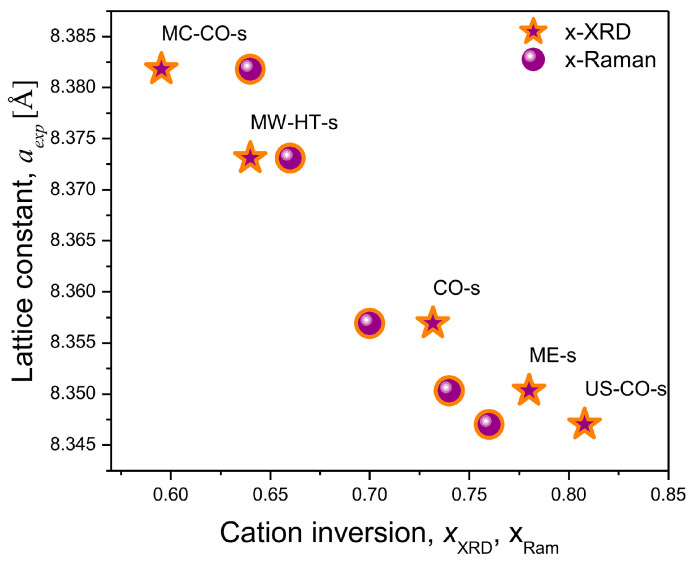
Comparison of inversion coefficients of starch-coated nanoparticles: Inversion coefficients obtained from XRD structural analysis (stars) and calculated from Raman spectra (circles) agree within Δ< ±0.05.

When compared with the inversion coefficients obtained on the basis of XRD structural analysis (stars) (Figure 8), it can be seen that *x*_R_ (circles) follows the trend of change, i.e., it gives a fairly good picture of the redistribution of cations in the starch-coated nanomaterial.

Spectral intensities and Lorentzian widths in deconvoluted Raman spectra can be associated with structural disorder. In the spectra of starch-coated NPs, the weak *F*_2g_ (3) mode is more clearly visible than in the spectra of bare NPs, regardless of whether their average size increased or decreased during the mixing process in the starch solution. It can be concluded that all coated nanopowders experienced some reduction in structural disorder.

The disappearance of the *A*_1g_* mode also confirms that the coating leads to a reduction in structural disorder. Indeed, in order to match the spectra of the bare MW-HT and MC-CO samples, it was necessary to introduce the *A*_1g_* mode, which corresponds to the maghemite-like depleted surface layer. After the starch coating process, these peaks disappear in both materials, and the intensity of the spectra becomes higher. The intensity of the MW-HT(s) spectrum is higher than that of the bare NPs, although the average size of the coated NP-s is slightly reduced according to XRD.

**Figure 9:**
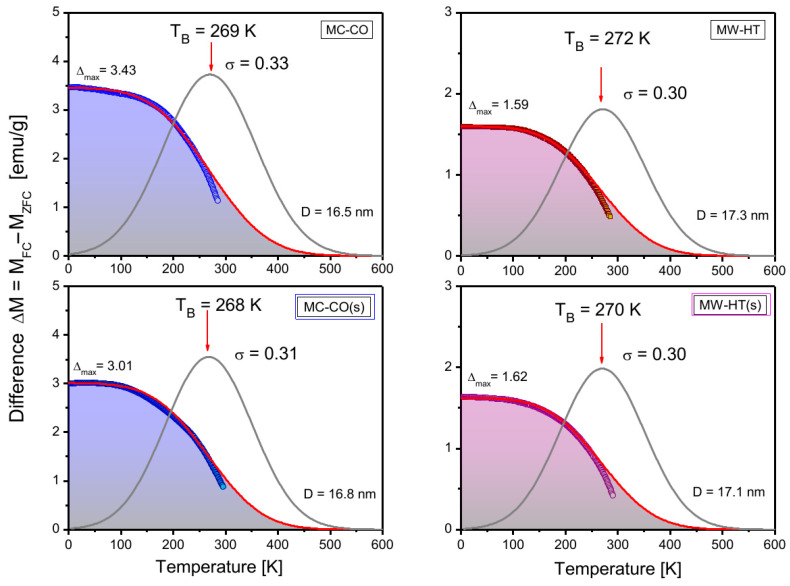
Fitting of difference in magnetizations, Δ*M(T)* = *M*_FC_ − *M*_ZFC_, was performed using FC/ZFC procedure. Fitting yields a distribution of blocking temperatures (*T*_B_), which corresponds to nanoparticle size distribution. The first row in the figure shows fitting results for pure nanoparticles MC-CO and MW-HT, while the second row shows fits for their coated counterparts. Circles represent experimental values, and red line represents theoretical fit Δ*M = f(T)* with a Gaussian distribution (grey line) of effective blocking temperatures, *T*_B_, and corresponding standard deviations. Resulting fitting parameters are shown in the figure.

The average size of nanoparticles in MC-CO(s) increases during the coating process. The size distribution becomes narrower, and the average size increases at the expense of the smallest nanoparticles. As the width of the blocking temperature (*T*_B_) distribution is equivalent to the width of the size distribution, narowing of size distribution is confirmed in Figure 9, which shows the results of fitting the *T*_B_ distribution for starch-coated and bare samples. The fitting model and procedure on the basis of the modified Stoner-Wohlfarth model [32,33] were applied in our previous work, Ref. [22], for uncoated samples.

Based on the data from Figure 9, a clear narrowing of the size distribution for the MC-CO(s) sample and a reduction in the Δ_max_ ∝ *1*/*K*_eff_ compared to bare MC-CO can be seen. Reduction of Δ_max_ in MC-CO(s) coincides with the increase of nanoparticle size. For the MW-HT(s) sample, with greater nanoparticles and an unchanged narrow size distribution, the changes in Δ_max_ are much smaller and accompanied by a slight decrease in nanoparticle size. The Raman spectra of these two samples show that the starch coating leads to a reduction in the magnetically depleted shell of the nanoparticles.

### 3.4. ^57^Fe-Mössbauer Spectroscopy

**Figure 10:**
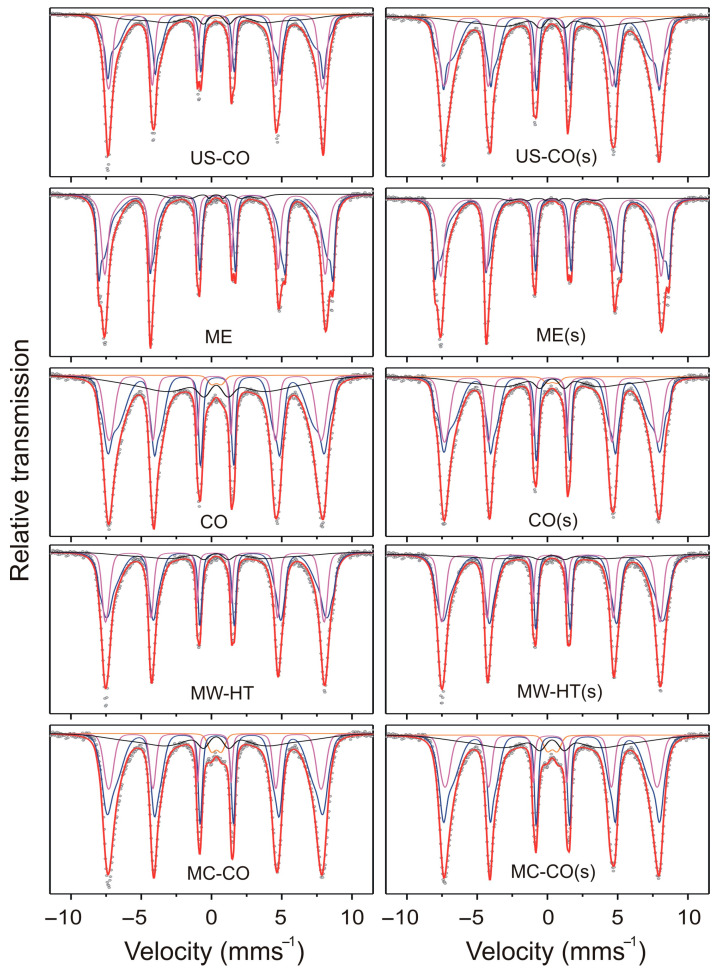
Room temperature ^57^Fe-Mössbauer spectra of bare and starch-coated CoFe_2_O_4_ nanoparticle samples. Experimental data are presented by solid circles, and Voigt-based fit is given by red solid line. Fitted Mössbauer sub-spectra are: QSD-site 1—orange doublet, HFD-site 1 (B sub-sites which resultant is shown as blue sextet), HFD-site 2 (A-site magenta sextet) and HFD-site 3 (black poorly defined sextet).

^57^Fe-Mössbauer spectroscopy is a perfect and efficient characterization technique to investigate the local structure of the Mössbauer-active element such as Fe. Spin behaviors of the bare, caped, core–shell iron oxide nanoparticles were discussed, based on the results of Mössbauer spectroscopy. In our previous work, the ^57^Fe-Mössbauer spectroscopy was used to investigate the pure CoFe_2_O_4_ nanoparticles prepared by different synthesis routes [34]. In this work, we investigated the same samples that were afterwards additionally coated with starch. The aim is to determine in which way the process of starch coating and the presence of starch on the surface of nanoparticles impact the structure and magnetic properties of CoFe_2_O_4_. The Mössbauer spectra of pure nanomaterials and the same nanomaterials with a starch coating are presented together in Figure 10 for easier comparison. The fitting results are shown in Table 4 and Table 5.

Mössbauer spectra shown in Figure 10 are analyzed by Voigt-based fitting method (VBF) to describe arbitrarily-shaped hyperfine parameter distributions [35]. The thickness corrections were performed and the results presented in Table 4 and Table 5 are for the thin-limit spectra. The relative peak areas (3:2:1:1:2:3) and the Lorentzian linewidth (HWHM = 0.097 mm s^−1^) were fixed during the fitting procedure. The background was not allowed to float in the fits. In the case of starch-coated nanoparticles, a correction was made for the mass of starch obtained by TGA.

Each spectrum is fitted with two magnetic generalized sites (two broadened sextets depicted by the hyperfine field distributions, HFDs) corresponding to the main tetrahedral (A-site) and octahedral (B-site) Fe and one weak sextet corresponding to the disordered spins of Fe atoms present at the surface of the nanoparticles (in the magnetically depleted shell). For samples with smaller average size of nanoparticles, additional paramagnetic generalized site (broadened doublet depicted by the quadrupole splitting distribution, QSD) was necessary to describe some of the Fe atoms that are in superparamagnetic (SP) surrounding. Due to cation distribution, we used four Gaussian-components for describing distribution of the effective hyperfine magnetic field of octahedral Fe and two Gaussian-components for the field of less sensitive tetrahedral Fe [36,37,38]. For the poorly defined weak sextet (HFD site 3), the quadrupole shift was set to zero as for the ideal cubic spinel [39] and the isomer shift was set to 0.33 mms^−1^ in the final fits. It was found that such a sextet best describes the part of each spectrum where the absorption lines strongly overlap.

The paramagnetic contribution to the magnetic measurements is visible in all samples, but for the Mössbauer experiment the critical diameter of the SP particles is much smaller: *D*_sp_ < 3.3–5.4 nm. *D*_sp_ can be calculated based on the data [22], for the anisotropy constant *K*_eff(300K)_ ≈ 5 J/m^3^ and by the Néel–Arrhenius law [40]: *τ_m_* = *τ*_o_ exp (*K*_eff_*·v*/(*k*_B_·*T*)), where the measurement time for the Mössbauer experiment is *τ_m_* = 10^−8^ s and the characteristic relaxation time is τ_o_ ~10^−9^–10^−12^ s. For magnetic measurements, *D*_sp_ is < 8 nm when *τ_m_* = 100 s [41]. Due to the much shorter measurement time, there are more blocked nanoparticles and a lower contribution from SP in Mössbauer measurements.

Assuming that the recoilless factors of the ^57^Fe nuclei located at different crystallographic sites are the same, the relative area (*S*) of the corresponding Mössbauer partial spectra is equal to the relative site population (Pop.): *S*_A_/*S*_B_ = PopA/PopB. Therefore, the relative proportion of Fe at the site is: *S*_A_/*S*_B_ = *x*/2 − *x*. The coefficient of cation inversion can now be estimated as follows:

*x* = 2·(*S*_A_/*S*_B_)/(1 + *S*_A_/*S*_B_) (10)

We first fitted the spectrum of the ME sample whose A- and B- sextets are not completely overlapped. The inversion coefficient calculated based on the population values of A and B sites (Equation (10)) agrees quite well with the inversion coefficient determined by XRD (within the margin of error of *x*_XRD_). In the preliminary fits of other samples, we started from ME values as fixed hyperfine parameters, only the areas of the generalized sites were to float. After this preliminary adjustment to the individual spectrum was done, we lifted some of the constraints. Finally, from the various accepted fits, we choose the ones that were in overall agreement with each other regarding physical meaning.

Table 4 shows that the maximum values of the average hyperfine magnetic field, <|*B*_hf_|>, were obtained for the ME and MW-HT samples with the largest nanoparticles. Samples with smaller nanoparticles, obtained by coprecipitation methods: CO, US-CO and MC-CO, show lower values of <|*B*_hf_|>. Thereby, the values of <|*B*_hf_|> for ^57^Fe B-site in both groups increase with the increase of the inversion coefficient. It is expected considering the increase of the superexchange interaction, J_AB_ due to increase in the number of Fe ions in A-site with stronger superexchange interaction: J(Fe^B^-O-Fe^A^) > J(Fe^B^-O-Co^A^) [37]. The values of <|*B*_hf_|> for the A-site show no clear dependence. This is also to be expected for relatively small nanoparticles, as the total J_AB_ of tetrahedral ^57^Fe changes significantly less with a change in the degree of inversion.

The CO and MW-HT samples exhibit the largest deviations between the degrees of inversion determined by Mössbauer analysis (*x*_Moss_) and those obtained from XRD analysis (*x*_XRD_). For the other samples, the differences are smaller. As mentioned above, the sextet overlap prevents a more accurate determination of the inversion coefficient.

The relative areas of the doublets in the Mössbauer spectra of the samples with the -smallest nanoparticles are very small (≈0.9–3.1%). It can be concluded that among the samples with visible doublets in the spectra, the US-CO nanoparticles have the narrowest size distribution, and the MC-CO nanoparticles have the broadest size distribution.

All samples had to be fitted with an additional, poorly defined sextet (HFD-site 3), which is obviously related to the magnetically disordered surface layer of the nanoparticles. The contribution of HDF-site 3 is significant in the spectra of the samples with smaller particles, while its relative area is minimal in ME sample. The volume percentages of the disturbed, magnetically depleted layers estimated for pure CoFe_2_O_4_ samples in our previous paper (Ref. [22], Table 5), are significantly higher than the effective area of HFD-site 3. As a reminder, the influence of the less magnetically disordered parts of the surface layers of the nanoparticles, the “shell”, is taken into account in the fitting of the Mössbauer spectra via additional Gaussian components for the distribution of the effective hyperfine magnetic fields of the A and B sites.

The ^57^Fe-Mössbauer spectra of the investigated starch-coated CoFe_2_O_4_ samples are shown in Figure 10 and the fitting parameters are listed in Table 5. The comparison of the raw data for the starch-coated and untreated CoFe_2_O_4_ samples shows that the spectra are basically very similar. Only slight changes in the parameter values could be recognized. In the coated nanoparticles, the electrostatic interaction of the polar starch molecules with the surface of the nanoparticles leads to a decrease in Fe spin disorder. The consequences, such as the decrease in line broadening and the increase in the strength of the HF fields, are as expected.

In the fits of all starch-coated CoFe_2_O_4_ samples, we started from the corresponding “non-coated” CoFe_2_O_4_ values as fixed ^57^Fe-Mössbauer parameters. As the first fitting step, only the areas of the generalized sites were allowed to float. After this adjustment to the individual starch-coated spectrum, we allowed some of the parameters to float. The first parameters that were lifted are related to the surface and shell of the nanoparticles, under the assumption that the starch may dominantly influence the outer part of nanoparticle. We have used the information from the XRD determined degree of inversion for the individual sample to constrain some of the Mössbauer parameters. Finally, from the various accepted fits, we choose the ones that were in overall agreement with each other regarding physical meaning. The presented fits due to the overlap of the central components are not unique.

Table 5 shows that the hyperfine parameters for the starch-coated samples are comparable to those of the uncoated samples. Changes are observed in the values of HF field parameters and in the area populations, especially in the area population of the doublets. The reduction of the doublet area, i.e., the SP site population (from 0.9%, 1.37% and 3.1% in the uncoated US-CO, CO and MC-CO samples to 0.35%, 1.13% and 2.8% in the coated counterparts, respectively) suggests that the strong mixing in the starch solution led to the dissolution of the smallest nanoparticles and to the narrowing of the size distribution of the nanoparticles.

In US-CO(s), the decrease of the measured effective magnetic ^57^Fe hyperfine fields and the increase of their standard deviation are observed, which is related to the decrease of the local magnetic order. These changes indicate the decrease in the average size of the nanoparticles after starch coating. Figure 10 clearly shows the broadened sextet peaks in US-CO(s) compared to the bare US-CO sample. The decrease in *D* was confirmed by XRD and magnetic measurements. It is possible that the effects of nanoparticle reduction somewhat offset the potential effects of starch coating. The decrease in the inversion coefficient is consistent with ∆*x*_XRD_.

The reduction in the standard deviation of the measured effective ^57^Fe HF magnetic fields in the ME(s) sample shows some increase in local magnetic order compared to ME.

In the case of CO(s), XRD and magnetic measurements confirmed that there was a reduction in nanoparticle size during the starch deposition process. Despite the size reduction, the decrease in the standard deviation of the HF field and the increase in all HF magnetic fields are registered in the CO(s) sample. These are exactly the changes in the fitting parameters of the Mössbauer spectrum that are expected when the starch is successfully deposited.

The fitting parameters of the MW-HT(s) spectra have changed slightly compared to the parameters of the bare counterpart MW-HT. The strength of the HF magnetic field in the surface layer increases significantly, i.e., the disorder decreases, and the coefficient of cation inversion decreases (which is accompanied by a slight increase in magnetization).

For the MC-CO(s) sample, the increase in the standard deviations *σ*(|*B*_hf_|) with decreasing HF fields in the A-site and the surface layer as well as the increase in the effective area of the HFD-site 3 indicate a decrease in the size of the coated nanoparticles. However, the increase in the HF field at the B-site does not fit into the picture of a decrease in particle size.

Such inconsistent changes in the parameters of coated and uncoated nanoparticles are the result of different methods of nanoparticle production. Differences in surface quality, shape, nanoparticle size, size distribution, internal stress, etc. can lead to very different results under identical conditions of the starch coating process.

Mössbauer spectroscopy confirmed the influence of starch coating on increasing the magnetic order and decreasing the internal stress by cross-referencing the data obtained by XRD and magnetic measurements.

## 4. Conclusions

Comprehensive characterization of starch-coated CoFe_2_O_4_ nanoparticles using XRD, magnetic measurement, Raman spectroscopy and ^57^Fe-Mössbauer spectroscopy reveals complex structural and magnetic modifications induced by the coating process, with starch deposition demonstrating sample-dependent effects that strongly correlate with the bare nanoparticle properties and synthesis methods. The structural changes are most pronounced for the smaller nanoparticles (US-CO, CO, MC-CO), where significant changes in lattice parameters, particle size and internal stress occur during coating. XRD structural analysis only in the case of the US-CO sample, with the smallest pure nanoparticles, shows a significant change in the inversion coefficient after starch coating: Δ*x*_XRD_ = − 0.05. The difference between the Mössbauer-derived inversion parameters for the same samples is similar: Δ*x*_Moss_ = −0.04, which corresponds to a slight increase in magnetization of the starch-coated US-CO(s). A slight increase in magnetization compared to bare samples was also measured in the starch-coated CO(s) and MW-HT(s), although it was not accompanied by a corresponding decrease in the cation inversion. For all three mentioned samples, the average nanoparticle size decreased during the starch coating process. Saturation magnetization is reduced in MC-CO(s) and ME(s) samples, again without corresponding change in the cation inversion and despite the increase of average nanoparticle size. Certain reduction of magnetization is expected due to obscuring effect of nonmagnetic starch. The seemingly contradictory increase in the saturated magnetization of starch-coated samples is a consequence of the reduction of disorder in the surface layer and the increase in the contribution of this layer to the total magnetization.

Depending on the synthesis method, i.e., the electronic structure and surface disorder of the original particles, surface coarsening, shape, average size, size distribution, etc., a vigorous mixing of nanoparticles in the starch solution can lead to change of the average size of nanoparticles and forming of new size distribution. It is possible that partially dissolution of nanoparticle aggregates and nanoparticles itself occurred, what can lead to decrease of nanoparticle’s average size. On the other hand, due to ultrasound-driven Ostwald ripening, the growth of larger particles can be promoted at the expense of smaller particles.

The changes in magnetic properties reflect both direct starch effects and indirect consequences of particle size modifications, with the starch functionalization showing a synthesis method-dependent behavior.

The reduction in surface disorder is consistently observed in all samples, as evidenced by the disappearance of maghemite-like surface phases in Raman spectra and improved magnetic order proved in Mössbauer analysis. The coating process leads to dissolution of the smallest superparamagnetic particles and a narrowing of the size distributions, while the coercivity systematically decreases by 2–30% for all coated samples, confirming a reduction in magnetic dipole interactions. The study shows that the effectiveness of starch coating depends critically on the initial properties of the nanoparticles and the synthesis method, with smaller particles undergoing more dramatic structural changes. While complete surface coverage remains a challenge, even partial coating significantly affects magnetic properties through reduced surface disorder and altered interparticle interactions, providing valuable insights into surface-magnetism relationships that could guide the development of future biocompatible materials requiring controlled magnetic responses.

## Figures and Tables

**Table 1:** Structural parameters of CoFe_2_O_4_ samples. Samples are labeled according to synthesis method (abbreviation defined earlier in the text), and starch-coated samples are marked with “(s)”. Structural parameters are calculated from diffractograms: lattice constant *a*_exp_, obtained from Rietveld’s diffractogram profile refinement and lattice constant *a*_311_ estimated from the strongest (311) Bragg reflection using Equations (1) and (2); *u*—oxygen positional parameter, *x*—coefficient of cation inversion, *x*_struct_—obtained by XRD-structure analysis, *D*—crystallite size and *e*_str—_internal strain obtained by Rietveld refinement and Williamson-H−all plot. *D*_311_ is calculated by Debye−Scherrer relation. In the last column is the agreement factor for Rietveld profile fitting (*R*_wp_—weighted profile).

Sample	*a*_exp_ [Å] (Δ*a*_exp_)	*a*_311_ [Å] (Δ*a*_311_)	$u^{\bar{3}m}$	*x*	*x*_struct_	*D*[nm] (Δ*D*)	*D*_311_[nm] (Δ*D*_311_)	*e*_str_· 10^−4^	*R*_wp_
US-CO US-CO(s) Δ	8.3407 8.3486 (+0.0079)	8.3511 8.3623 (+0.0112)	0.2540 0.2543	0.80 0.85	0.85 0.81	15.80 15.10 (−0.7)	15.64 13.98 (−1.66)	21.0 17.8	19.5 14.89
ME ME(s) Δ	8.3503 8.3503 (0)	8.3650 8.3646 (−0.0004)	0.2545 0.2545	0.80 0.80	0.79 0.78	19.00 19.60 (+0.6)	22.87 22.96 (+0.09)	5.78 3.8	16.4 19.5
CO CO(s) Δ	8.3619 8.3569 (−0.0050)	8.3719 8.3654 (−0.0065)	0.2548 0.2548	0.78 0.75	0.73 0.73	16.47 14.55 (−1.92)	17.46 15.39 (−2.07)	9.1 12.9	21.79 17.51
MW-HT MW-HT(s) Δ	8.3732 8.3732 (0)	8.3741 8.3766 (+0.0025)	0.2554 0.2554	0.67 0.70	0.64 0.64	17.34 17.11 (−0.24)	17.33 17.03 (−0.30)	16.8 15.6	16.77 16.38
MC-CO MC-CO(s) Δ	8.3825 8.3875 (+0.0050)	8.3825 8.3938 (+0.0112)	0.2558 0.2557	0.59 0.60	0.58 0.59	16.46 16.79 (+0.33)	15.36 16.22 (+0.86)	20.0 15.3	16.3 16.7

**Table 2:** Magnetization measurements in magnetic field ± 90 kOe: saturation magnetization *M*_s_ at 90 kOe, absolute error Δ ≈ ±0.1 emu/g; extrapolated saturation magnetization at *H* = 0, *M*_sH=0_; coercivity, *H*_c_ (Δ_max_ = ±5 Oe); magnetic remanence, *M*_r_ (Δ ≈ ±0.1 emu/g); “squareness”, *M*_r_/*M*_s_ (Δ ≈ ±0.01); slope of magnetization in near-zero field region (d*M*/d*H*)_H=0_ and slope of magnetization in high field region (d*M*/d*H*)_H⟶90 kOe_, for starch-coated samples obtained by various synthesis methods. For comparison, values of corresponding magnetization slopes for bare samples are given. Samples are listed in ascending order of coercivity.

Samples	*M*_s_, (n) * [emu g^−1^], [μ_B_]	*M*_sH*=0*_ [emu g^−1^]	*H*_c_ [Oe]	*M*_r_ [emu g^−1^]	*M*_r_/*M*_s_	(d*M*/d*H*)_H=0_ [emu g^−1^/kOe]		(d*M*/d*H*)_H_⟶_90kOe_ [emu g^−1^/kOe]	
						Starch	Bare	Starch	Bare
CO (s)	69.9 (2.93)	66.1	231	11.0	0.16	52.86	46.7	0.0426	0.048
US-CO (s)	66.0 (2.77)	64.03	265	12.6	0.19	51.95	43.7	0.0222	0.037
MC-CO (s)	59.0 (2.48)	56.61	400	11.5	0.19	27.64	36.6	0.0265	0.035
MW-HT (s)	72.9 (3.06)	68.64	702	17.8	0.24	29.78	29.7	0.0473	0.045
ME (s)	62.6 (2.63)	58.94	1219	22.47	0.36	21.48	28.4	0.0410	0.046

n * = 234.623·*M*_s_/5585.

**Table 3:** Magnetic measurement data: saturation magnetization *M*_s_ at 300 K and *H* = 90 kOe, coercivity *H*_c_ and magnetization *M*_FC_ at 5 K and *H* = 100 Oe for pure CoFe_2_O_4_ nanoparticle samples and their starch-coated counterparts. Data for pure NPs are reprinted from our previous study [22]. Δ is difference between measured values (in parenthesis). In the last column, average nanoparticle size *D* determined using Rietveld refinement is added (rewritten from Table 1 to make it easier to see dependence of *H*_c_ and *M*_FC_ with *D*).

Sample	*M*_s_ [emu g^−1^], [μ_B_] (Δ*M*_s_)/%	*H*_c_ [Oe] (Δ*H*_c_)/%	*M*_FC_ [emu g^−1^] (Δ*M*_FC_)/%	*D* [nm] (Δ*D*)
US-CO US-CO(s) Δ	61.0 (2.56) 66.0 (2.77) (+5.0)/7%	291 265 (−26)/−9%	4.05 4.45 (+0.35)	15.80 15.10 (−0.7)
ME ME(s) Δ	69.3 (2.91) 62.6 (2.63) (−6.7)/−11%	1206 1219 (+13)/1%	1.2 0.8? (−0.4)	19.00 19.60 (+0.6)
CO CO(s) Δ	66.3 (2.78) 69.9 (2.94) (+3.6)/5%	330 231 (−99) −43%	3.95 5.50 (+1.55)	16.47 14.55 (−1.92)
MW-HT MW-HT(s) Δ	70.0 (2.94) 72.9 (3.06) (+2.9)/4%	723 702 (−21)/−3%	1.75 1.76 (+0.01)	17.34 17.11 (−0.24)
MC-CO MC-CO(s) Δ	72.4 (3.04) 59.0 (2.48) (−13.4)/−23%	407 400 (−7)/−2%	3.57 3.1 (−0.47)	16.46 16.79 (+0.33)

**Table 4:** Room temperature ^57^Fe-Mössbauer hyperfine parameters for **pure** CoFe_2_O_4_ nanoparticles: HFD—hyperfine magnetic field distribution, QSD—quadrupole splitting distribution; Site Pop.—site populations; <*CS*>—center shift; <|Δ|>—centroid (average) of quadrupole splitting distribution; *σ* (|Δ|)—standard deviation of quadrupole splitting distribution (Gaussian width); <*ε*>—quadrupole shift in case of combined strong magnetic and weak electric interaction; <|*B*_hf_|>—centroid (average) of hyperfine magnetic field distribution; *σ*(|*B*_hf_|)—standard deviation of hyperfine magnetic field distribution (Gaussian width); **—multicomponent fit for effective hyperfine magnetic field; QSD-site 1, broadened doublet; HFD-site 1, large broadened sextet B-site; HFD-site 2, broadened sextet A-site; HFD-site 3, poorly defined sextet.

Sample		<*CS*> [mm s^−1^]	<ε> [mm s^−1^]	<|*B*_hf_|> [T]	<|Δ|> [mm s^−1^]	*σ* (|*B*_hf_|) [T]	*σ* (|Δ|) [mm s^−1^]	Site Pop. [%]	*x*_MOSS_ (*x*_XRD_)
US-CO	QSD-site 1 SP-doublet	0.35			0.7		0.4	0.903 (88)	0.86 (0.85)
	HFD-site 1 [B site]	0.37	−0.069	45.9 **		3.5		47.93 (67)	
	HFD-site 2 (A site)	0.24	0.047	47.3 **		1.4		36.45 (52)	
	HFD-site 3 Shell	0.33	0	35.3		9.9		14.72 (85)	
ME	HFD-site 1 [B site]	0.37	−0.064	48.7 **		3.8		57.20 (78)	0.82 (0.79)
	HFD-site 2 (A site)	0.23	0.02	48.1 **		2.7		39.68 (77)	
	HFD-site 3 Shell	0.33	0	19.2		3.1		3.12 (27)	
CO	QSD-site 1 SP-doublet	0.35			0.7		0.4	1.37 (17)	0.67 (0.73)
	HFD-site 1 [B site]	0.37	−0.034	45.6 **		4.1		51.20 (98)	
	HFD-site 2 (A site)	0.23	0.037	46.8 **		1.9		25.62 (84)	
	HFD-site 3 Shell	0.33	0	33.1		15		21.81 (90)	
MW-HT	HFD-site 1 [B site]	0.39	−0.015	47.6 **		3.7		58.4 (14)	0.69 (0.64)
	HFD-site 2 (A site)	0.22	0.019	48.0 **		1.3		30.8 (10)	
	HFD-site 3 Shell	0.33	0	33.5		11.3		10.8 (17)	
MC-CO	QSD-site 1 SP-doublet	0.35			0.7		0.4	3.137 (44)	0.60 (0.58)
	HFD-site 1 [B site]	0.31	−0.064	45.8 **		3.6		55.36 (72)	
	HFD-site 2 (A site)	0.22	0.02	46.9 **		1.8		23.76 (54)	
	HFD-site 3 Shell	0.33	0	35.1		12.8		17.74 (78)	

**Table 5:** Room temperature ^57^Fe-Mössbauer hyperfine parameters for the **starch-coated** CoFe_2_O_4_ nanoparticles: HFD—hyperfine magnetic field distribution, QSD—quadrupole splitting distribution; Site Pop.—site populations; <*CS*>—center shift; <|Δ|>—centroid (average) of quadrupole splitting distribution; *σ*(|Δ|)—standard deviation of quadrupole splitting distribution (Gaussian width); <*ε*>—quadrupole shift in case of combined strong magnetic and weak electric interaction; <|*B*_hf_|>—centroid (average) of hyperfine magnetic field distribution; *σ*(|*B*_hf_|)—standard deviation of hyperfine magnetic field distribution (Gaussian width); **—multicomponent fit for effective hyperfine magnetic field; QSD-site 1, broadened doublet; HFD-site 1, large broadened sextet B-site; HFD-site 2, broadened sextet A-site; HFD-site 3, poorly defined sextet. Arrows show change in value relative to bare counterpart.

Sample		<*CS*> [mm s^−1^]	<*ε*> [mm s^−1^]	<|*B*_hf_|> [T]	<|Δ|> [mm s^−1^]	*σ*(|*B*_hf_|) [T]	*σ*(|Δ|) [mm s^−1^]	Site Pop. [%]	*x*_MOSS_ (*x*_XRD_)
US-CO(s)	QSD-site 1 SP-doublet	0.35			0.7		0.4	0.35(15) ↓	0.81 ↓ (0.81)
	HFD-site 1 [B site]	0.35 ↓	−0.068 ↓	45.8 ** ↓		4.1 ↑		51.1(15) ↑	
	HFD-site 2 (A site)	0.25 ↑	0.044 ↓	47.1 ** ↓		2.0 ↑		34.8(12) ↓	
	HFD-site 3 Shell	0.33	0	33.5 ↓		11.4 ↑		13.7(16) ↓	
ME(s)	HFD-site 1 [B site]	0.37	−0.053↓	48.8 **↑		3.5 ↓		58.34(67) ↑	0.82 (0.78)
	HFD-site 2 (A site)	0.23	0.02	48.1 **		2.4 ↓		40.31(66) ↑	
	HFD-site 3 Shell	0.33	0	18.7 ↓		1.8 ↓		1.35(25) ↓	
CO(s)	QSD-site 1 SP-doublet	0.35			0.7		0.4	1.13(15) ↓	0.70↑ (0.73)
	HFD-site 1 [B site]	0.36 ↓	−0.043	45.8 ** ↑		3.9 ↓		55.4(14) ↑	
	HFD-site 2 (A site)	0.24 ↑	0.039 ↑	47.0 **↑		1.9		29.8(11)↑	
	HFD-site 3 Shell	0.33	−0.043	33.7 ↑		11.5 ↓		13.6(14)↓	
MW-HT(s)	HFD-site 1 [B site]	0.39	−0.015	47.4 ** ↓		3.7		61.1(13) ↑	0.67↓ (0.64)
	HFD-site 2 (A site)	0.22	0.020	48.0 **		1.3		30.74(82) ↓	
	HFD-site 3 Shell	0.33	0	34.5↑		12.5 ↑		8.2(16) ↓	
MC-CO(s)	QSD-site 1 SP-doublet	0.35			0.7		0.4	2.81(16) ↓	0.61↑ (0.59)
	HFD-site 1 [B site]	0.34 ↑	−0.044↓	45.9 ** ↑		3.6		54.99(84) ↓	
	HFD-site 2 (A site)	0.22	0.04↑	46.8 ** ↓		1.9 ↑		23.90(56) ↑	
	HFD-site 3 Shell	0.33	0	33.4 ↓		13.4 ↑		18.30(95) ↑	

## Data Availability

The original contributions presented in this study are included in the article material. Further inquiries can be directed to the corresponding authors.
